# Multi-omics analysis reveals a novel *NFE2L3* variant impairing choroidal vasculature development in high myopia and myopic maculopathy

**DOI:** 10.1093/nsr/nwae291

**Published:** 2024-08-29

**Authors:** Jiangnan He, Luyao Ye, Hannan Xu, Huanjie Yang, Juan Shen, Menghan Li, Shijun Weng, Dongyue Jiao, Chen Chu, Qijun Liao, Haidong Zou, Jianfeng Zhu, Chenji Wang, Xun Xu

**Affiliations:** Department of Preventative Ophthalmology, Shanghai Eye Diseases Prevention &Treatment Center/Shanghai Eye Hospital, School of Medicine, Tongji University, National Clinical Research Center for Eye Diseases, Shanghai Engineering Research Center of Precise Diagnosis and Treatment of Eye Diseases, China; Department of Ophthalmology, Shanghai General Hospital, Shanghai Jiao Tong University School of Medicine, Shanghai Clinical Research Center for Eye Diseases, Shanghai Key Clinical Specialty, Shanghai Key Laboratory of Ocular Fundus Diseases, Shanghai Engineering Center for Visual Science and Photomedicine, China; Department of Ophthalmology, Shanghai General Hospital, Shanghai Jiao Tong University School of Medicine, Shanghai Clinical Research Center for Eye Diseases, Shanghai Key Clinical Specialty, Shanghai Key Laboratory of Ocular Fundus Diseases, Shanghai Engineering Center for Visual Science and Photomedicine, China; BGI Research, China; College of Life Sciences, University of Chinese Academy of Sciences, China; BGI Research, China; Institute of Metagenomics, Qingdao-Europe Advanced Institute for Life Sciences, BGI Research, China; Department of Ophthalmology, Shanghai General Hospital, Shanghai Jiao Tong University School of Medicine, Shanghai Clinical Research Center for Eye Diseases, Shanghai Key Clinical Specialty, Shanghai Key Laboratory of Ocular Fundus Diseases, Shanghai Engineering Center for Visual Science and Photomedicine, China; State Key Laboratory of Medical Neurobiology and MOE Frontiers Center for Brain Science, Institutes of Brain Science, Fudan University, China; State Key Laboratory of Genetic Engineering, MOE Engineering Research Center of Gene Technology, Shanghai Engineering Research Center of Industrial Microorganisms, School of Life Sciences, Fudan University, China; Department of Preventative Ophthalmology, Shanghai Eye Diseases Prevention &Treatment Center/Shanghai Eye Hospital, School of Medicine, Tongji University, National Clinical Research Center for Eye Diseases, Shanghai Engineering Research Center of Precise Diagnosis and Treatment of Eye Diseases, China; BGI Research, China; Department of Preventative Ophthalmology, Shanghai Eye Diseases Prevention &Treatment Center/Shanghai Eye Hospital, School of Medicine, Tongji University, National Clinical Research Center for Eye Diseases, Shanghai Engineering Research Center of Precise Diagnosis and Treatment of Eye Diseases, China; Department of Ophthalmology, Shanghai General Hospital, Shanghai Jiao Tong University School of Medicine, Shanghai Clinical Research Center for Eye Diseases, Shanghai Key Clinical Specialty, Shanghai Key Laboratory of Ocular Fundus Diseases, Shanghai Engineering Center for Visual Science and Photomedicine, China; Department of Preventative Ophthalmology, Shanghai Eye Diseases Prevention &Treatment Center/Shanghai Eye Hospital, School of Medicine, Tongji University, National Clinical Research Center for Eye Diseases, Shanghai Engineering Research Center of Precise Diagnosis and Treatment of Eye Diseases, China; State Key Laboratory of Genetic Engineering, MOE Engineering Research Center of Gene Technology, Shanghai Engineering Research Center of Industrial Microorganisms, School of Life Sciences, Fudan University, China; Department of Preventative Ophthalmology, Shanghai Eye Diseases Prevention &Treatment Center/Shanghai Eye Hospital, School of Medicine, Tongji University, National Clinical Research Center for Eye Diseases, Shanghai Engineering Research Center of Precise Diagnosis and Treatment of Eye Diseases, China; Department of Ophthalmology, Shanghai General Hospital, Shanghai Jiao Tong University School of Medicine, Shanghai Clinical Research Center for Eye Diseases, Shanghai Key Clinical Specialty, Shanghai Key Laboratory of Ocular Fundus Diseases, Shanghai Engineering Center for Visual Science and Photomedicine, China

Myopic maculopathy (MM) is a severe complication of high myopia, characterized by degenerative lesions in the macular retina, choroid and sclera [1]. The pooled prevalence of MM is estimated to be 2.1% in the world population and 47.4% among highly myopic adults [2]. It poses significant challenges due to its poor prognosis and limited treatment options, and has become a major cause of irreversible visual impairment worldwide [3,4]. Notwithstanding, comprehensive investigations into the genetic implications of high myopia and MM remain insufficient. To bridge this gap, we conducted a case–control association study in Chinese individuals, integrating whole-exome sequencing (WES) to identify potential causal genes and investigate their roles in the pathogenesis of high myopia and MM (Fig. S1).

We first analysed a WES data set from the Shanghai High Myopia Study for Adults, which included 697 Han Chinese individuals. Among them, 403 were highly myopic patients with MM, carefully chosen for their representativeness, while the remaining 294 were emmetropic controls with high education levels (Table S1). After the variants were filtered and annotated (Tables S2 and S3), a total of 763 251 coding variants were subjected to further analysis. These included 108 520 common variants (minor allele frequency [MAF] ≥ 5%) and 654 731 low-frequency variants (MAF < 5%). Inflation factor analysis indicated similar genetic backgrounds between the cases and controls (λ = 1.05, Fig. S2a). A total of 726 common variants reached suggestive significance (*P* < 0.005, Fig. S2b and Table S4). Also, we performed six nested gene-based analyses for low-frequency variants that exhibit larger per-allele effect sizes than common variants and offer crucial biological insights (Fig. S2c) [5]. Four genes, namely *PRR35, ATP2B4, NFE2L3* and *LAMA5*, were identified as reaching suggestive significance (odds ratio > 2 and *P* < 0.005) based on a deleterious (strict) set (Fig. 1a, Fig. S2d and Table S5). The most significant leading variants for these four candidate genes are listed (Fig. 1b) and the genome location and protein structure of variants are illustrated (Fig. 1c, Figs S2e and S3a–f). Collectively, the WES analysis reveals four causal genes associated with an increased risk of high myopia and MM.

**Figure 1.**
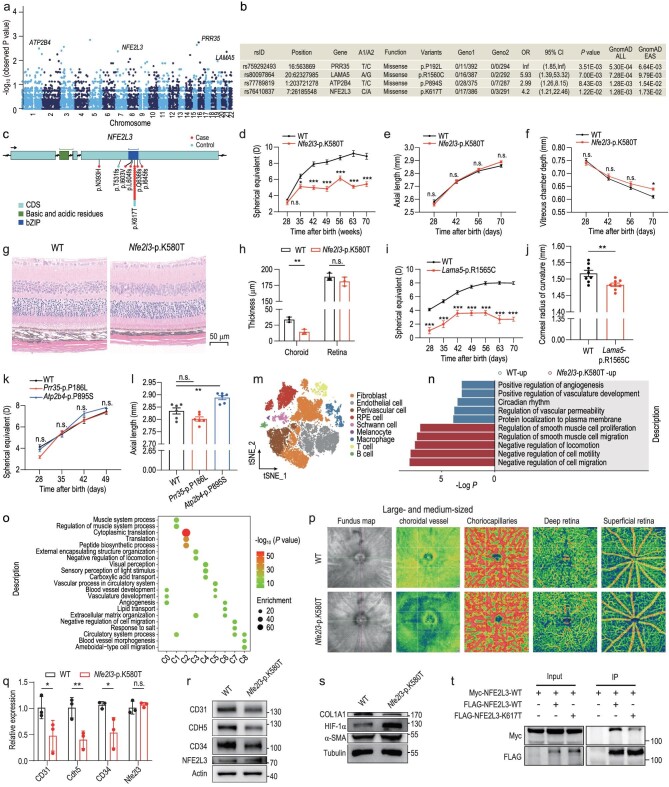
A novel *NFE2L3* variant identified in highly myopic patients with myopic maculopathy leads to myopia in knock-in mice by impairing choroidal vasculature development. (a) Manhattan plot presenting the results of gene-based associations based on a deleterious (strict) set (odds ratio > 2 and *P* < 0.005). (b) The most significant leading variants in *PRR35, ATP2B4, NFE2L3* and *LAMA*5 based on gene-based analysis in a deleterious (strict) set. A1 = minor allele; A2 = major allele; Geno1 = the genotype count for the homozygous minor, heterozygous and homozygous major alleles in cases; Geno2 = the genotype count for the homozygous minor, heterozygous and homozygous major alleles in controls; CI = confidence interval; OR = odds ratio; gnomAD ALL = variant allele frequency in the general population obtained from Genome Aggregation Database; gnomAD EAS = variant allele frequency in the East Asian population obtained from Genome Aggregation Database. (c) Variants identified in whole-exome sequencing data for *NFE2L3*. (d)–(f) The development of (d) refractive error, (e) axial length and (f) vitreous chamber depth of *Nfe2l3*-p.K580T and wild-type (WT) mice from P28 to P70 (*n* = 8/group). (g) and (h) Hematoxylin and eosin staining of *Nfe2l3*-p.K580T and WT mice at (g) P70 and (h) the quantification of choroidal thickness and retinal thickness (*n* = 3/group). Scale bars, 50 μm. (i) The development of refractive error in *Lama5*-p.R1565C and WT mice from P28 to P70 (*n* = 8/group). (j) The corneal radius of curvature of *Lama5*-p.R1565C and WT mice at P70 (*n* = 8/group). (k) and (l) The development of the (k) refractive error and (l) axial length of *Atp2b4*-p.P895S and *Prr35*-p.P186L mice from P28 to P49 (*n* = 6/group). (m) T-distributed Stochastic Neighbor Embedding representation of single-cell gene expression showing nine identified choroidal cell types. RPE = retinal pigment epithelial. (n) Gene ontology (GO) enrichment of the differentially expressed genes identified in endothelial cells between *Nfe2l3*-p.K580T and WT choroids. (o) GO enrichment of the top 200 highly expressed genes identified in each subpopulation of endothelial cells. (p) Visualization of fundus map, large and medium-sized choroidal vessels blood flow, choriocapillaris blood flow, deep retinal blood flow and superficial retinal blood flow in *Nfe2l3*-p.K580T and WT mice using OCT-A (*n* = 12/group). Capture view size: 12 mm × 12 mm. (q) Quantitative real-time reverse transcription polymerase chain reaction for messenger ribonucleic acid expression (*n* = 3/group) and (r) Western blot for the protein expression of CD31, CDH5 and CD34 in *Nfe2l3*-p.K580T and WT choroids (*n* = 3/group). (s) Western blot for the protein expression of HIF-1α, α-SMA and COL1A1 in *Nfe2l3*-p.K580T and WT sclera (*n* = 3/group). (t) Western blot analysis of the whole-cell lysates and immunoprecipitates from 293 T cells transfected with the indicated plasmids. Error bars represent standard error of the mean. n.s. = not significant. *P < 0.05; **P < 0.01; ***P < 0.001. Paired *t*-test or Student's *t*-test.

To assess the potential impact of these missense variants on refraction development, we generated four strains of systemic knock-in (KI) mice (Fig. S4a–d and Table S6). Compared with wild-type (WT) mice, *Nfe2l3*-p.K580T (homologous to p.K617T in humans) mice displayed myopic refractive shift from P35 to P70 (all *P* < 0.05; Fig. 1d). Ocular biometric measurements revealed a trend for longer axial length (AL) without statistical significance (2.86 ± 0.03 versus 2.89 ± 0.02 mm; *P* = 0.33; Fig. 1e) and significantly longer vitreous chamber depth (VCD; 0.61 ± 0.02 versus 0.64 ± 0.01 mm; *P* = 0.02; Fig. 1f) in *Nfe2l3*-p.K580T mice at P70. The anterior chamber depth, lens thickness and corneal radius of curvature (CRC) were similar between the two groups (Fig. S5a–c). Additionally, hematoxylin and eosin (H&E) staining demonstrated that *Nfe2l3*-p.K580T mice exhibited significantly thinner choroidal thickness but indistinguishable retinal thickness compared with WT mice at P70 (Fig. 1g and h). *Lama5*-p.R1565C (homologous to p.R1560C in humans) mice exhibited myopia from P28 to P70 compared with WT mice (all *P* < 0.001; Fig. 1i). There were no significant differences in axial components between the two groups (Fig. S5d–g) but the CRC of *Lama5*-p.R1565C mice was significantly smaller than that of WT mice at P70 (1.517 ± 0.022 versus 1.483 ± 0.011 mm; *P* = 0.007; Fig. 1j). H&E staining and optical coherence tomography imaging showed normal retinal structure of *Lama5*-p.R1565C mice at P70 (Fig. S5h–j). However, transmission electron microscopy revealed disrupted membrane discs in both *Nfe2l3*-p.K580T and *Lama5*-p.R1565C mice (Fig. S5k). Refraction and ocular biometric measurements indicated that *Atp2b4*-p.P895S (homologous to p.P894S in humans) and *Prr35*-p.P186L (homologous to p.P192L in humans) mice displayed comparable ocular development to WT mice, except that *Atp2b4*-p.P895S mice had a significantly longer AL (2.83 ± 0.02 versus 2.89 ± 0.02 mm; *P* = 0.004) (Fig. 1k and l). Collectively, we validate that *Nfe2l3*-p.K580T and *Lama5*-p.R1565C mice displayed myopic refractive shifts that were attributed to elongated VCD and smaller CRC, respectively.

Given that neither *Atp2b4*-p.P895S nor *Prr35*-p.P186L mice displayed a myopic phenotype, and the reduced CRC observed in *Lama5*-p.R1565C mice did not coincide with the continuous axial elongation seen in humans with high myopia and MM, we focused our investigations on discerning the mechanisms underlying the myopic phenotype observed in *Nfe2l3*-p.K580T mice. Considering the thinner choroidal thickness observed in the mutant mice, which is a typical characteristic during the development and progression of high myopia and MM [6], we conducted single-cell ribonucleic acid sequencing (scRNA-seq) on choroid samples from *Nfe2l3*-p.K580T and WT mice at P70 (Table S7). By analysing the expression of markers and referring to recent scRNA-seq studies of choroids [7], we identified nine distinct cell types (Fig. 1m, Fig. S6a–c and Table S8). Enrichment analysis using the differentially expressed genes within each subcluster was conducted on gene ontology (Table S9). The 49 downregulated genes in endothelial cells (ECs) were enriched in processes related to the positive regulation of angiogenesis, vasculature development and vascular permeability. Conversely, the 43 upregulated genes in ECs were enriched in processes such as the negative regulation of locomotion, cell mobility and cell migration (Fig. 1n and Fig. S6d). We further identified eight transcriptionally distinct subpopulations of ECs (Fig. S6e). Subpopulations that were predominant in WT mice (C0, C2 and C5) demonstrated enrichment in genes associated with blood vessel development, vasculature development and angiogenesis. In contrast, subpopulations that were predominant in *Nfe2l3*-p.K580T mice (C1, C3 and C7) were associated with extracellular matrix organization and negative regulation of locomotion and cell migration (Fig. 1o and Fig. S6f). These results indicated a potential association between ECs in *Nfe2l3*-p.K580T mice and the negative regulation of vasculature development.

To further validate the choroidal vascular dysfunction in *Nfe2l3*-p.K580T mice, we performed an *in vivo* experiment involving optical coherence tomography angiography (OCT-A) imaging of the fundus (Fig. 1p). Quantitative analysis confirmed a reduction in blood flow within large and medium-sized choroidal vessels, choriocapillaris and deep retina in the *Nfe2l3*-p.K580T group compared with the control group (Fig. S7a–c). No significant difference was found in superficial retinal blood flow layers between the two groups (Fig. S7d). These results indicated a decreased blood flow perfusion in the choroid of *Nfe2l3*-p.K580T mice. Furthermore, quantitative real-time reverse transcription polymerase chain reaction and Western blot analyses revealed decreased expression of vascular EC markers in the choroids of *Nfe2l3*-p.K580T mice (Fig. 1q–r and Fig. S7e and f). In the sclera of *Nfe2l3*-p.K580T mice, an elevated expression of HIF-1α and a decreased expression of COL1A1 were observed (Fig. 1s and Fig. S7g). Collectively, these findings indicate that the *Nfe2l3*-p.K580T variant impairs choroidal vascularization and causes scleral hypoxia and extracellular matrix remodeling [8], contributing to the development of myopia in mice.

The *NFE2L3*-p.K617T, identified as a potential pathogenic candidate, was exclusive to the East Asian population (Fig. S8a). Its origins can be traced back by ∼44 503 years, which corresponds to 1780 generations (Fig. S8b). NFE2L3 is a transcription factor belonging to the CNC-bZIP family [9], with the bZIP domain playing a crucial role in dimerization and DNA binding [10]. Most of the variants of NFE2L3 found in the WES data were located in the bZIP domain (Fig. 1c). We examined the interaction affinity between FLAG- and Myc-tagged NFE2L3 proteins. Compared with the FLAG-NFE2L3-WT, less Myc-NFE2L3 was immunoprecipitated by FLAG-NFE2L3-p.K617T (Fig. 1t). These results indicated that variants in the bZIP domain, specifically p.K617T, hinder the dimerization of NFE2L3. This impairment of dimerization may have negative effects on the function of NFE2L3 as a transcription factor.

In conclusion, we have identified a novel variant, *NFE2L3*-p.K617T, that increases the risk of high myopia and MM in Chinese heterozygous carriers using WES and demonstrated its deleterious roles using KI mice models. Mechanistic investigations revealed that this variant impairs choroidal vasculature development, leading to decreased choroidal blood flow perfusion, ultimately resulting in scleral hypoxia and myopia development. Although this low-frequency variant may only explain a portion of the phenotypic variance, our findings have enhanced the understanding of the biological mechanisms involved in high myopia and MM, and provide insights for potential therapeutic targets.

## Supplementary Material

nwae291_Supplemental_Files
