# Supplementary material for: Multi-omics analysis reveals a novel NFE2L3 variant impairing choroidal vasculature development in high myopia and myopic maculopathy
Source: Natl Sci Rev. 2024 Aug 29;12(2):nwae291. doi: 10.1093/nsr/nwae291 (PMC11879437; doi:10.1093/nsr/nwae291)
Supplement: nwae291_Supplemental_Files [file nwae291_supplemental_files.zip › Supplementary_Materials-R2.doc]

Supplementary Information for

**Multi-omics analysis reveals a novel *NFE2L3* variant impairing choroidal vasculature development in high myopia and myopic maculopathy**

Jiangnan He^1^*, Luyao Ye^2,8^*, Hannan Xu^2^*, Huanjie Yang^5,6^*, Juan Shen^5,7^*, Menghan Li^2^, Shijun Weng^4^, Dongyue Jiao^3^, Chen Chu^1^, Qijun Liao^6^, Haidong Zou^1,2^, Jianfeng Zhu^1^, Chenji Wang^3^, Xun Xu^1,2^

*^1^Department of Preventative Ophthalmology, Shanghai Eye Diseases Prevention &Treatment Center/Shanghai Eye Hospital, School of Medicine, Tongji University, National Clinical Research Center for Eye Diseases, Shanghai Engineering Research Center of Precise Diagnosis and Treatment of Eye Diseases, Shanghai, China*

*^2^Department of Ophthalmology, Shanghai General Hospital, Shanghai Jiao Tong University School of Medicine, Shanghai Clinical Research Center for Eye Diseases, Shanghai Key Clinical Specialty, Shanghai Key Laboratory of Ocular Fundus Diseases, Shanghai Engineering Center for Visual Science and Photomedicine, Shanghai, China*

*^3^State Key Laboratory of Genetic Engineering, MOE Engineering Research Center of Gene Technology, Shanghai Engineering Research Center of Industrial Microorganisms, School of Life Sciences, Fudan University, Shanghai, China*

*^4^State Key Laboratory of Medical Neurobiology and MOE Frontiers Center for Brain Science, Institutes of Brain Science, Fudan University, Shanghai, China.*

*^5^BGI Research, Sanya, China*

*^6^College of Life Sciences, University of Chinese Academy of Sciences, Beijing, China*

*^7^Institute of Metagenomics, Qingdao-Europe Advanced Institute for Life Sciences, BGI Research, Qingdao, China*

*^8^Luyao Ye’s current affiliation is Department of Ophthalmology, The Affiliated People's Hospital of Ningbo University, Ningbo, China*

*These authors contributed equally.

**Correspondence** Xun Xu (drxuxun@sjtu.edu.cn) and Chenji Wang ([chenjiwang@fudan.edu.cn](mailto:chenjiwang@fudan.edu.cn))

**The PDF file includes:**

Materials and Methods

Supplementary References

Author contributions and acknowledgements

Supplementary Figures S1 to S8

Supplementary Tables S1 to S3, S5 to S7, and S10

Other Supplementary Tables are listed as separate files including Tables S4, S8, and S9

**Materials and Methods**

**Study population**

This study included 412 highly myopic adults with myopic maculopathy (MM) who were recruited from the Shanghai High Myopia Study for Adults (SHMSA) at Shanghai Eye Diseases Prevention and Treatment Center in Shanghai, China. Individuals aged ≥18 years, with spherical power ≤−6.00 diopter (D) or axial length (AL) ≥26 mm in either eye were invited to register. The inclusion criteria for MM patients were based on the META-PM classification system^1^, which was used for diagnosis in this study. The control group comprised 298 individuals who participated in ophthalmic screenings in communities or colleges. The inclusion criteria for the control group were as follows: 1) age ≥ 18 years; 2) -0.25 D ≤ spherical power ≤ +2.0 D; 3) uncorrected visual acuity ≥ 20/25; 4) education background of at least a college degree. Exclusion criteria for both cohorts included previous intraocular or refractive surgery, corneal opacity, severe cataract, glaucoma, retinal pathology nonrelated to high myopia (e.g., diabetic retinopathy, age-related macular degeneration, and optic neuropathy), severe systemic diseases, secondary myopia, any familial relationships with the enrolled participants, and poor-quality images. All participants underwent standardized clinical interviews and comprehensive ophthalmic examinations, including measurement of AL using an optical low-coherence reflectometry (Lenstar LS-900; Haag-Streit AG), assessment of spherical equivalents using an autorefractor (model KR-8900; Topcon), slit-lamp biomicroscopy, and color fundus examination with swept-source optical coherence tomography (OCT, model DRI Atlantis; Topcon). A summary description of the samples used in this study (after whole-exome sequencing quality control) is provided in **[Table S1](https://www.ncbi.nlm.nih.gov/pmc/articles/PMC8387277/#mmc1)**. The study protocol was approved by the Ethics Committee of Shanghai General People’s Hospital, Shanghai, China (Approval number: 2015KY156), in accordance with principles of the Declaration of Helsinki. Written informed consent forms were obtained from all participants.

**Whole-exome sequencing**

The procedures for DNA extraction, purification, fragmentation, hybridization, library construction, and sequencing were carried out following well-established protocols and methods^2^. In brief, DNA was extracted from the peripheral blood using the Magnetic Universal Genomic DNA kit (TIANGEN). High-quality genomic DNA samples were fragmented using Covaris technology, yielding library fragments with lengths ranging between 200-300 bp. Subsequently, these DNA fragments underwent hybridization with the SureSelectXT Human All Exon V6 exome array (Agilent) for targeted enrichment of specific genomic regions. The enriched libraries were then sequenced on BGISEQ-500 platforms (BGI) to facilitate high-throughput sequencing. Ultimately, the sequencing process yielded an average depth of approximately 174.96× with paired-end 100 bp reads.

**Alignment and Variant Calling**

To ensure the integrity of data, the initial processing involved filtering raw reads to eliminate adapters and low-quality sequences with SOAPnuke (v2.1.0)^3^. Subsequently, the high-quality reads were aligned to the GRCh38 reference genome using Burrows-Wheeler Aligner (BWA v0.7.17)^4^. Duplicated reads were identified and removed using Genome Analysis Toolkit (GATK v4.1.4.1)^5^. To further enhance the accuracy and precision of variant calling, local realignment around insertions and deletions (indels) was performed, and base quality scores were recalibrated on the resulting BAM files using GATK. Single nucleotide variants and small indels were identified utilizing GATK HaplotypeCaller and genotyped jointly through the GATK GenotypeGVCFs tool, following recommended best-practice guidelines^6^.

**Variants and sample quality control**

Regarding the variants, we implemented a stringent filtering strategy to retain only highly reliable variants. This approach involved the following steps: 1) GATK hard-filtering; 2) Ensuring a mean sequencing depth of variants for both cases and controls ≥ 8×; 3) Requiring a mean depth for the minor allele ≥ 4×; 4) Mandating a minimum read coverage of 8 reads in ≥ 90% of samples containing the variant; 5) Ensuring a root mean square of mapping quality ≥ 30; 6) Strand bias: FS < 5 and SOR < 3; 7) Allelic balance between 0.25 and 0.75; 8) Homopolymer runada < 6; 9) Hardy-Weinberg equilibrium test P ≥ 10^-6^. Post filtration, 763,251 variants remained, and these were further annotated using ANNOVAR^7^.

To ensure high sample quality, a series of stringent measures were applied to exclude samples with lower confidence. These criteria included: 1) Average sequencing depth: < 30×; 2) Coverage distribution: less than 90% of the genome covered at a minimum of 10×; 3) Identification of samples indicating a 3rd degree or closer relationship; 4) GC content: < mean - standard deviation or > mean + standard deviation; 5) Mapping rate: < 95%; 6) Inbreeding coefficient: > 0.15 or < -0.15; 7) Principal Component Analysis (PCA): > 6σ from the mean of the first 10 principal components; 8) Sex determination: chrY coverage rate > 0.8 for males and < 40% for females. Following these quality control measures, a total of 403 cases and 294 controls were retained for subsequent analysis.

**Single marker association analysis**

PCA was performed utilizing the PLINK software (version 1.9) on the genetic dataset obtained from all patients^8^. This analytical approach aimed to condense the data dimensions and unveil prominent patterns of genetic diversity. Following this, logistic regression analysis was conducted using PLINK to investigate prevalent genetic variants (with minor allele frequency [MAF] > 5%) potentially associated with high myopia and MM. The primary focus involved employing the top 10 principal components derived from the PCA as covariates in the regression analysis to mitigate any influence from population stratification and potential confounding factors.

**Gene-based association analysis**

Gene-based collapsing analyses are particularly well effective in identifying a clear link between the causal gene and phenotype^9^. Five protein prediction algorithms (LRT score, MutationTaster, PolyPhen-2 HumDiv, PolyPhen-2 HumVar, and SIFT) were applied on missense variants to identify the most probable low-frequency damaging variants (MAF < 5%). Then, six variant sets were defined, as follows: 1) A “Nonsyn” set: nonsense and missense variants; 2) A “deleterious (broad)” set: nonsense, splice-site, indel frameshift, and missense variants annotated as “deleterious” by at least one protein prediction algorithm; 3) A “deleterious (polyphen)” set: nonsense, splice-site, indel frameshift, and missense variants annotated as “deleterious” by “PolyPhen-2 HumDiv” algorithm; 4) A “deleterious (strict)” set: nonsense, splice-site, indel frameshift, and missense variants annotated as “deleterious” by all five protein prediction algorithms; 5) “LOF” variants set: nonsense, splice-site, and indel frameshift variants; 6) “Frameshift” variants set: indel frameshift variants. Subsequently, the collapse method was used with the two-tailed Fisher's exact test in R 3.6.1 software to perform gene-based association analysis on six defined variant sets.

**Knock-in mice generations**

Mutant knock-in mice were designed and generated by Shanghai Model Organisms Center. In brief, Atp2b4-p.P895S, Lama5-p.R1565C, and Nfe2l3-p.K580T heterozygous knock-in mouse lines were generated by zygote injection of CRISPR/Cas9 constructs and a single-stranded oligo donor DNA through homology-directed repair. The guide RNA sequences used are listed in **Table [S6](https://www.ncbi.nlm.nih.gov/pmc/articles/PMC7608219/#MOESM11)**. Prr35-p.P186L mouse line was generated by blastocyst injection of transgenic JM8A3 C57BL/6 embryonic stem cells. Mice were housed under a 12-hour light:12-hour dark cycle with an ambient illumination of approximately 200 lux during the daytime, and provided with abundant food and water. All animal experiments were conducted in accordance with protocols approved by the Institutional Animal Care and Use Committee of Shanghai Institute of Biochemistry and Cell Biology.

### Refractive error and ocular biometric measurements

Refractive error was assessed using an eccentric infrared photorefractor (Steinbeis Transfer Center)^10^. Before measurement, the mice were administered with tropicamide and phenylephrine hydrochloride solution (Mydrin-P, Santen Pharmaceutical) to dilate pupils. The measurements were conducted without anesthesia in a dark environment. Three sets of refraction data were recorded for each eye and averaged. All mice were screened at P28, wherein those with an initial interocular refractive difference greater than 2 D or exhibiting abnormal pupil images were excluded from subsequent experiments.

Axial components were measured using a custom-made, ultra-long depth, spectral domain-OCT (SD-OCT) system with a resolution of 10 μm, as previously described^11^. Briefly, the mice were scanned under anesthesia using a combination of 0.6% pentobarbital and 0.2% xylazine (10 μl/g body weight). The raw SD-OCT images were exported and analyzed using a custom-designed software to derive an averaged value for the axial components, including anterior chamber depth (the distance from the posterior corneal surface to the anterior lens surface), lens thickness (the distance from the anterior lens surface to the posterior lens surface), vitreous chamber depth (the distance from the posterior lens surface to the vitreous-retina interface), and AL (the distance from the anterior corneal surface to the vitreous-retina interface).

Corneal radius of curvature was measured using a keratometer equipped with a +20 D achromatic doublet lens (OM-4, Topcon), following previously described methods^11^. Briefly, mice were measured under anesthesia, and the mean value from the three measurements was used for further comparisons and analysis.

**Fundus blood flow Imaging**

Fundus blood flow imaging was performed using an ultra-widefield OCT and OCT angiography system (BM-400 K BMizar, Toward Pi Medical Technology)^12^. This system utilized a long-wavelength (1060 nm) full range swept source and a 400 kHz A-scan rate. The mice were dilated after intraperitoneal anesthesia and high-resolution scan images of the various layers of the fundus centered on optic disc were captured. The retinal vessels and choriocapillaris were identified using higher-order moments decorrelation algorithm. The large- and medium-sized choroid vessels were visualized by a 3-dimentional threshold segmentation algorithm. The average blood flow density of the large- and medium-sized choroidal vessels, choriocapillaris, deep retinal vessels, and superficial retinal vessels were then evaluated within a 12 mm × 12 mm tomographic scan area.

**Hematoxylin and eosin staining**

Three right eyes from Nfe2l3-p.K580T, Lama5-p.R1565C, and wild-type (WT) mice at P70 were enucleated and fixed in 10% neutral buffered formalin overnight. The fixed eyes were then paraffin-embedded, axial sectioned at a thickness of 5 μm, and subjected to Hematoxylin and eosin staining. Three measurements of retinal thickness and choroidal thickness were taken per section and averaged, approximately 300 μm from the optic nerve.

**Transmission electron microscopy**

Three left eyes from Nfe2l3-p.K580T, Lama5-p.R1565C, and WT mice at P70 were enucleated and fixed overnight in TEM Fixative (Servicebio). The samples were fixed for 1 hour in 1% osmium tetroxide, dehydrated using a graded ethanol series, and embedded in epon 812 resin. Ultra-thin sections containing retina were block-stained with 2% uranium acetate saturated alcohol solution and 2.6% lead citrate. These sections were then examined using a transmission electron microscope (HT7800/HT7700; HITACHI).

**Single-cell RNA sequencing sample collection, initial processing, library preparation, and sequencing**

Nfe2l3-p.K580T and WT mice at P70 were euthanized through cervical dislocation. Both eyes were then enucleated, and the choroids were isolated for the preparation of a single cell suspension. Due to insufficient yields of cells from individual choroidal tissues, 6 mice (12 eyes) were pooled in each group and collected in RPMI-1640 medium (Gibco). The collected choroids were gently enzymatically digested using a Tumor Dissociation Kit (Miltenyi Biotec) following the manufacturer’s protocol. The digested cells were then filtered using a 40-um nylon mesh, along with [Red Blood Cell](https://www.sciencedirect.com/topics/medicine-and-dentistry/erythrocyte) Lysis Solution (Meltenyi Biotec) and the Dead Cell Removal Kit (Miltenyi Biotec). For single-cell RNA sequencing (scRNA-seq) libraries, the Chromium Next GEM Single Cell 3ʹ Library and Gel Beads Kit v3.1 (10x Genomics) was used following the manufacturer’s instructions. The concentration of cDNA and DNA libraries for each sample was assessed using the [Qubit](https://www.sciencedirect.com/topics/medicine-and-dentistry/quantum-dot) 4 Fluorometers (Thermo), and quality control was performed using the Agilent 4150 TapeStation (Agilent Technologies). Finally, the single-cell transcriptomes were sequenced on the Ilumina Novaseq 6000 platform, generating 150 bp paired-end reads (conducted by OE Biotech).

**Pre-processing of scRNA-seq data**

Alignment to the mouse genome mm10, quantification, and preliminary quality checks were performed using the 'cellranger' pipeline (v7.0.1) from 10X Genomics^13^. The 'mkfastq' function was utilized to generate fastq files from raw Illumina BCL files. These fastq files were then used with the 'count' function to obtain read count matrices. Subsequently, the 'aggr' function was applied to aggregate all scRNA-seq libraries without depth normalization, resulting in an expression matrix.

The resulting expression matrix was imported into Seurat objects via the R package 'Seurat' (v4.3.0.1) (https://github.com/satijalab/seurat). Quality control measures were implemented to remove outlier cells and genes^14^. Cells were filtered based on the following criteria: 1) cells with fewer than 600 genes; 2) cells with more than 7500 genes; 3) cells with over 20% unique molecular identifiers derived from mitochondrial genes; 4) potential doublets identified by the DoubletFinder algorithm (v2.0.3)^15^; 5) low abundance genes with fewer than 3 detected cells. After applying these quality control standards, 28,384 single cells were retained for downstream analyses.

Following the removal of unwanted cells and genes, the data was normalized and dimensionality reduction was performed. The feature expression measurements for each cell were normalized by the total expression, and the inherent variation was regressed using the 'ScaleData' function (vars.to.regress = c("percent.mt", "nCount_RNA", "orig.ident")). Next, the top 5000 highly variable genes were selected for PCA dimensionality reduction. To correct for batch effects, Harmony (v0.1.1) was used on the first 50 principal components^16^. Subsequently, the Louvain algorithm-based clustering was applied to generate cell clusters, using the 'FindNeighbors' and 'FindClusters' functions with optimized 'resolution' values for each analysis. Finally, the clustering results were visualized using t-distributed Stochastic Neighbor Embedding.

**Identification of marker genes and cell type annotation**

Cell types were annotated manually based on cluster-specific markers. The Seurat 'FindAllMarkers' function (only.pos = TRUE, min.pct = 0.25, logfc.threshold = 0.25) was used to identify marker genes for each cell type. Marker genes previously identified in choroidal scRNA-seq studies were listed as follows^17-19^: fibroblasts (Mgp, Dcn, Lum, Cdh11, Col1a1), ECs (Cdh5, Pecam1, Vwf, Cd34), perivascular cells (Cspg4, Acta2, Myh11), retinal pigment epithelial cells (Rpe65, Rlbp1), Schwann cells (Ncmap, Cdh19, Nrn1, Sox10, Plp1, Prnp, Mbp, Mag, Mpz), Melanocytes (Mlana, Tyrp1), Macrophages (Cd74, Cd14, Cd83, Aif1), T cells (Cd3d, Cd3e), and B cells (Igkc, Cd79a, Ms4a1). Furthermore, Gene Ontology (GO) enrichment of marker genes for each cell type was performed using Metascape (http://metascape.org)^20^.

**Identification of differentially expressed genes**

To identify genes that showed differential expression between mutant and WT mice across different cell types in the single-cell dataset, differential gene expression analysis was conducted using Seurat's FindMarkers function with the parameter test.use = "wilcox". Subsequently, GO enrichment analysis on the identified differentially expressed genes was employed using Metascape (http://metascape.org)^20^.

### Real-time quantitative PCR

### Total RNA was extracted from the choroid of two eyes of mice using the RNA pure Micro Kit following the manufacturer’s protocol (JIANSHI Biotech). 800ng of RNA was used for reverse transcription, employing the RT Master Mix (Vazyme). Quantitative real-time PCR was carried out using the SYBR Green qPCR SuperMix (Vazyme), utilizing the ViiA™ 7 Real-Time PCR System (Applied Biosystems). A reference gene validation experiment was conducted to determine the most stably transcribed housekeeping gene in the choroid tissue. Four commonly used reference genes were selected from the literature for validation^21^: *Gapdh* ([glyceraldehyde](https://baike.baidu.com/item/glyceraldehyde/24434816?fromModule=lemma_inlink)-3-phosphate dehydrogenase), *Actb* (β-Actin), *Hprt* (hypoxanthine phosphoribosyltransferase), and *Rpl13a* (ribosomal protein L13a). Gapdh was found to be transcribed most stably between *Nfe2l3*-p.K580T and WT choroids and was chosen as a reference gene. The primers used are presented in Table S6. Relative gene mRNA expressions were determined using the 2^−ΔΔCt^ method, with normalization to Gapdh.

**Western blot**

# Choroid samples from two eyes were lysed using RIPA buffer and quantified using the Bradford reagent (Beyotime). Equal amounts of freshly extracted lysate were separated by electrophoresis on 4-20% SDS-PAGE gels. The proteins were then transferred onto polyvinylidene fluoride membrane (Roche) using the Transfer-Blot Turbo Transfer System (Bio-Rad). To block non-specific binding, the membranes were incubated in TBS-T (TBS + 0.1% Tween-20) with 5% nonfat milk powder at 37 °C for 1 h. Subsequently, the membranes were incubated overnight at 4°C with primary antibodies, which are listed in Table S10. Following this, a secondary antibody (Jackson Immuno Research Labs, diluted 1:5000) was applied and incubated at room temperature for 1 hour. Finally, the signals were visualized using the ChemiDoc Imaging System (Bio-Rad).

**Plasmids and mutagenesis**

Constructs of *NFE2L3* and its mutants were generated in our laboratory. The cDNAs of p62 were obtained from Genecopia Inc. The cDNAs were subcloned into pCMV-FLAG or Myc vector. *NFE2L3* mutants were generated by KOD-Plus-Mutagenesis Kit (TOYOBO) following the manufacturer’s instructions.

**Cell lines, culture, and transfection**

293T cells was purchased from American Type Culture Collection, which was cultured in Dulbecco’s modified Eagle’s medium supplemented with 10% FBS. The cells were maintained in a 37 °C humidified incubator supplied with 5% CO_2_. For transient transfection, the cells were transfected with Lipofectamine 2000 (Thermo) according to the manufacturer’s instructions.

### Co-immunoprecipitation (Co-IP)

293T cells were transfected with indicated constructs. 36 h after transfection, cells were lysed in 1% SDS buffer (Tris pH 7.5, 0.5 mM EDTA, 1 mM DTT) and boiled for 10 minutes. For immunoprecipitation, the cell lysates were diluted 10-fold in Tris-HCl buffer and incubated with anti-FLAG M2 agarose beads for 4 h at 4 °C. The bound beads are then washed four times with BC100 buffer (20 mM Tris-Cl, pH 7.9, 100 mM NaCl, 0.2 mM EDTA, 20% glycerol) containing 0.2% Triton X-100. The protein was eluted with 3×FLAG peptide for 2 h at 4 °C. The dimerization was detected by Western blot using anti-Myc antibody.

**Statistical analysis**

The results are presented as the mean ± standard error of the mean. The difference between groups was analyzed using either paired *t* test or Student’s *t* test. GraphPad Prism 9 and SPSS 26.0 were used for all statistical analyses.

**Data availability**

The variation data reported in this paper has been deposited in the Genome Variation Map^22^ in National Genomics Data Center^23^, China National Center for Bioinformation/Beijing Institute of Genomics, Chinese Academy of Sciences (accession number GVM000572, http://bigd.big.ac.cn/gvm/getProjectDetail?project=GVM000572). To request access, contact the Lead Author.

**Supplementary References**

1. Ohno-Matsui K, Kawasaki R, Jonas JB et al. International photographic classification and grading system for myopic maculopathy. *Am J Ophthalmol* 2015; 159: 877-883.

2. Yao K, Dai Y, Shen J et al. Exome sequencing identifies rare mutations of LDLR and QTRT1 conferring risk for early-onset coronary artery disease in Chinese. *Natl Sci Rev* 2022; 9: nwac102.

3. Chen Y, Chen Y, Shi C et al. SOAPnuke: a MapReduce acceleration-supported software for integrated quality control and preprocessing of high-throughput sequencing data. *Gigascience* 2018; 7: 1-6.

4. Li H, Durbin R. Fast and accurate short read alignment with Burrows-Wheeler transform. *Bioinformatics* 2009; 25: 1754-1760.

5. McKenna A, Hanna M, Banks E et al. The Genome Analysis Toolkit: A MapReduce framework for analyzing next-generation DNA sequencing data. *Genome Res* 2010; 20: 1297-1303.

6. Ulintz PJ, Wu W, Gates CM. Bioinformatics Analysis of Whole Exome Sequencing Data. *Methods Mol Biol* 2019; 1881: 277-318.

7. Wang K, Li M, Hakonarson H. ANNOVAR: functional annotation of genetic variants from high-throughput sequencing data. *Nucleic Acids Res* 2010; 38: e164.

8. Chang CC, Chow CC, Tellier LC et al. Second-generation PLINK: rising to the challenge of larger and richer datasets. *Gigascience* 2015; 4: 7.

9. Povysil G, Petrovski S, Hostyk J et al. Rare-variant collapsing analyses for complex traits: guidelines and applications. *Nat Rev Genet* 2019; 20: 747-759.

10. Zhu X, Du Y, Li D et al. Aberrant TGF-beta1 signaling activation by MAF underlies pathological lens growth in high myopia. *Nat Commun* 2021; 12: 2102.

11. Liu AL, Liu YF, Wang G et al. The role of ipRGCs in ocular growth and myopia development. *Sci Adv* 2022; 8: eabm9027.

12. Gui SY, Tang WW, Huang ZH, et al. Ultrasmall Coordination Polymer Nanodots Fe-Quer Nanozymes for Preventing and Delaying the Development and Progression of Diabetic Retinopathy. *Adv Funct Mater* 2023; 33: 202300261.

13. Zheng GX, Terry JM, Belgrader P et al. Massively parallel digital transcriptional profiling of single cells. *Nat Commun* 2017; 8:14049.

14. Hao Y, Hao S, Andersen-Nissen E et al. Integrated analysis of multimodal single-cell data. *Cell* 2021;184: 3573-3587.

15. McGinnis CS, Murrow LM, Gartner ZJ. DoubletFinder: Doublet Detection in Single-Cell RNA Sequencing Data Using Artificial Nearest Neighbors. *Cell Syst* 2019; 8: 329-337.

16. Korsunsky I, Millard N, Fan J et al. Fast, sensitive and accurate integration of single-cell data with Harmony. *Nat Methods* 2019; 16: 1289-1296.

17. Lehmann GL, Hanke-Gogokhia C, Hu Y et al. Single-cell profiling reveals an endothelium-mediated immunomodulatory pathway in the eye choroid. *J Exp Med* 2020; 217: e20190730.

18. Voigt AP, Mulfaul K, Mullin NK et al. Single-cell transcriptomics of the human retinal pigment epithelium and choroid in health and macular degeneration. *Proc Natl Acad Sci U S A* 2019; 116: 24100-24107.

19. Gautam P, Hamashima K, Chen Y et al. Multi-species single-cell transcriptomic analysis of ocular compartment regulons. *Nat Commun* 2021; 12: 5675.

20. Zhou Y, Zhou B, Pache L et al. Metascape provides a biologist-oriented resource for the analysis of systems-level datasets. *Nat Commun* 2019; 10:1523.

21. Alizadeh P, Smit-McBride Z, Oltjen SL, et al. Regulation of cysteine cathepsin expression by oxidative stress in the retinal pigment epithelium/choroid of the mouse. *Exp Eye Res* 2006; 83: 679-87.

22. Li C, Tian D, Tang B et al. Genome Variation Map: a worldwide collection of genome variations across multiple species. *Nucleic Acids Res* 2021; 49: D1186-D1191.

23. CNCB-NGDC Members and Partners. Database Resources of the National Genomics Data Center, China National Center for Bioinformation in 2023. *Nucleic Acids Res* 2023; 51: D18-D28.

**Author contributions**

X.X, C.J.W, and J.N.H conceptualized the study.

L.Y.Y drafted the manuscript.

L.Y.Y and H.N.X revised the manuscript.

L.Y.Y, H.N.X, M.H.L, S.J.W, D.Y.J, and C.C contributed to animal experiments.

J.S., H.J.Y, and Q.J.L analyzed the whole-exome sequencing and single-cell data.

J.N.H, J.F.Z, and H.D.Z were responsible for sample collection.

X.X and J.N.H were responsible for funding acquisition.

X.X, C.J.W, and J.N.H supervised the project.

All authors reviewed and edited the manuscript.

**Acknowledgements**

This study was supported by the National Key Research and Development Program of China (Project No. 2022YFC2502800, 2019YFC0840607, 2022YFA1104200, 2022YFC2703102), the National Natural Science Foundation of China (Grant No: 82171100, 91957125, 32370726, 82304089), Shanghai Key Disciplines to strengthen the construction of Public health system Three-year action Plan (2023-2025) (GWVI-11.1-22), Shanghai Shenkang Hospital Clinical Research Program (Project No. SHDC12020127). The sponsors or funding organizations had no role in the design or conduct of this research.

**Supplementary Figures**

**Supplementary Figure 1.** General design of the study. We identify candidate genes of high myopia and myopic maculopathy using whole-exome sequencing data, validate the pathogenicity of the identified variants by constructing knock-in mice, and reveal the possible pathogenesis using single-cell RNA sequencing and mechanism investigations. The Figure was partly generated using Servier Medical Art, provided by Servier, licensed under a Creative Commons Attribution 3.0 unported license. VCD = vitreous chamber depth; WT = wild-type.

**Supplementary Figure 2. Single marker and gene-based association results by whole-exome sequencing.**

**a** Q-Q plot illustrating the association test statistics in the whole-exome sequencing study. **b** Manhattan plot displaying the results of single marker association for common variants. **c** Q-Q plot depicting the nested gene-based analyses in six sets: (1) Nonsyn set; (2) deleterious (broad) set; (3) deleterious (polyphen) set; (4) deleterious (strict) set; (5) LOF variants set; (6) frameshift variants set. **d** Forest map illustrating the nested gene-based analyses conducted for *PRR35*, *ATP2B4*, *NFE2L3,* and *LAMA5* across six sets. OR = odds ratio; CI = confidence interval. **e** Three-dimensional structure of NFE2L3 with the location of recurrent variants indicated using Alphafold DB.

**Supplementary Figure 3****.** **The distribution of variants in *PRR35, ATP2B4,* and *LAMA5.***

**a** Variants identified in the whole-exome sequencing data for *PRR35*, *ATP2B4*, and *LAMA5*. Red dots represent cases, while blue dots represent controls. **b** Three-dimensional structure of PRR35, ATP2B4, and LAMA5 with the location of recurrent variants indicated using Alphafold DB or RCSB PDB.

**Supplementary Figure 4. Constructions and validations of mutant knock-in mice.**

**a** Schematic illustrating the construction strategy for knock-in mouse models of *Atp2b4*, *Lama5*, and *Nfe2l3* using the CRISPR/Cas9 system. In brief, zygotes of C57BL/6 mice were injected with Cas9 mRNA, guide RNA, and single-stranded oligo donor DNA to generate heterozygous F0 mice. **b** Schematic presenting the construction strategy for the *Prr35* knock-in mouse model using embryonic stem cell targeting. JM8A3 C57BL/6 embryonic stem cells were electroporated with a targeting vector consisting of a 2.9-kb 5′ arm, a PGK-Neo-polyA positive selection cassette, a fragment of mutant *Prr35* exon 2, a 3.0-kb 3′ arm, and a MC1-TK-polyA negative selection cassette. Positive clones were identified through long-range PCR, expanded, and then injected into C57BL/6 blastocysts to generate chimeric mice. Heterozygous F1 mice were generated by crossing the chimeric mice with Flp recombinase-expressing mice. **c** Alignment of genomic DNA and protein sequences of human and mouse near the variant locus. The lysine (K) residue at position 617 in human NFE2L3 is equivalent to lysine 580 in mouse NFE2L3. Likewise, the arginine (R) residue at position 1560 in human LAMA5 corresponds to arginine 1565 in mouse LAMA5. Additionally, the proline (P) residue at position 894 in human ATP2B4 is equivalent to proline 895 in mouse ATB2B4. Similarly, the proline (P) at position 192 in human PRR35 corresponds to proline 186 in mouse PRR35. d Validation of homozygous knock-in mice through Sanger sequencing.

**Supplementary Figure 5. The ocular biometric components and fundus examination of Nfe2l3-p.K580T and Lama5-p.R1565C mice.**

**a-b** The anterior chamber depth (**a**) and lens thickness (**b**) of Nfe2l3-p.K580T and wild-type (WT) mice were measured from P28 to P70 (n=8/group). **c** The corneal radius of curvature in Nfe2l3-p.K580T and WT mice at P70 (n=8/group). **d-g** The axial length (**d**), vitreous chamber depth (**e**), anterior chamber depth (**f**), and lens thickness (**g**) of Lama5-p.R1565C and WT mice were measured from P28 to P70 (n=8/group). **h-i** Hematoxylin and eosin staining was performed on Lama5-p.R1565C and WT mice at P70 (**h**) and the choroidal thickness and retinal thickness were quantified (**i**, n=3/group). **j** An ultra-widefield swept-source optical coherence tomography was used to visualize the fundus structure of Nfe2l3-p.K580T, Lama5-p.R1565C, and WT mice at P70 (n=12/group)*.* **k** Transmission electron microscope was used to examine the photoreceptor of outer segment of Nfe2l3-p.K580T, Lama5-p.R1565C, and WT mice at P70 (n=3/group)*.* Error bars represent standard error of the mean. n.s., not significant. Paired t test or Student’s t test.

**Supplementary Figure 6. Sc-RNA seq analysis of choroids from Nfe2l3-p.K580T and wild-type mice.**

**a** The marker genes for the nine identified choroidal cell types. RPE = retinal pigment epithelial. **b** Color-coded for the expression of the indicated marker genes. Scale: white/gray is low expression; red is high gene expression. **c** Gene ontology enrichment of the marker genes identified in each choroidal cell type. **d** Volcano plot displaying differentially expressed genes (DEG) of endothelial cells between Nfe2l3-p.K580T and wild-type (WT) choroids. **e** T-distributed Stochastic Neighbor Embedding representation of single-cell gene expression of endothelial cells showing eight identified subpopulations. **f** Comparison of the distribution of eight identified endothelial cells subpopulations between Nfe2l3-p.K580T and WT mice.

**Supplementary Figure 7*.* Nfe2l3-p.K580T mice exhibit decreased choroidal blood flow perfusion and scleral hypoxia.**

**a-d** Quantification of blood flow density in the large- and medium-sized choroidal vessels (**a**), choriocapillary (**b**), deep retina (**c**), and superficial retina (**d**) layers of the fundus. **e** Quantification of the mRNA expression of *Gapdh*, *Actb*, *Hprt*, and *Rpl13a* in *Nfe2l3*-p.K580T and wild-type (WT) choroids for reference gene selection (n = 3/group). **f** Quantification of the protein expression of CD31, CDH5, and CD34 in Nfe2l3-p.K580T and WT choroids (n=3/group). **g** Quantification of the protein expression of HIF-1α, α-SMA, and COL1A1 in Nfe2l3-p.K580T and WT sclera (n=3/group). Error bars represent standard error of the mean. n.s., not significant. *P < 0.05, ** P < 0.01, *** P < 0.001. Student’s t test.

**Supplementary Figure 8*. NFE2L3*-p.K617T allele frequencies and estimated emergence time across different populations.**

**A** The allele frequency information of *NFE2L3*-p.K617T in various populations from 1000G, ExAC, and gnomAD databases. ALL: All populations, EUR: European, EAS: East Asian, SAS: South Asian, AFR: African/African American, AMR: Admixed American; FIN: Finnish, NFE: Non-Finnish European, ASJ: Ashkenazi Jewish, OTH: Other (population not assigned). **B** Data and figure sourced from https://human.genome.dating indicate the presence of *NFE2L3*-p.K617T in human populations dating back to 1780 generations age, which is equivalent to approximately 44,503 years (with a generation span of 25 years). TGP: the 1000 Genomes Project; SGDP: the Simons Genome Diversity Project."

**Supplementary Tables**

**Supplementary Table 1. Baseline characteristics of patients with myopic maculopathy and control participants**

| **Characteristics** | **MM** | **Control** |
| --- | --- | --- |
|  | **(*N* = 403)** | **(*N* = 294)** |
| Age, year | 66.90 (9.48) | 38.81 (18.12) |
| Male, n (%) | 159 (39.45) | 106 (36.05) |
| Spherical equivalent, D* | -13.49 (4.61) | -0.06 (0.75) |
| Axial length, mm | 29.40 (1.82) | 23.04 (0.82) |
| BCVA, logMAR^†^ | 0.62 (0.40) | 0.00 (0.07) |
| MM catergory |  |  |
| Category 1 (tessellated fundus) | 7 (1.74) | 0 |
| Category 2 (diffuse atrophy) | 224 (55.58) | 0 |
| Category 3 (patchy atrophy) | 104 (25.81) | 0 |
| Category 4 (macular atrophy) | 67 (16.63) | 0 |
| Lacquer cracks | 246 (61.04) | 0 |
| Myopic CNV/Fuchs spot | 70 (17.37) | 0 |

BCVA = best-corrected visual acuity; CNV = choroidal neovascularization; D = diopter; LogMAR = logarithm of minimal angle of resolution; MM = myopic maculopathy. The continuous variables were described as mean ± standard deviation, and the categorical variables were listed as number (percentage). * 65 pseudophakic eyes at baseline were excluded from the calculation of mean spherical equivalent. ^†^ 33 eyes with counting fingers, hand motion, light perception, or no light perception at baseline were excluded from the calculation of mean BCVA.

**Supplementary Table 2. Summary of whole exome sequencing across all individuals before quality control**

|  | **MM** | **Control** |
| --- | --- | --- |
|  | **(*N* = 412)** | **(*N* = 298)** |
| Total effective bases (Mb) | 18746.90 | 15866.79 |
| Effective sequences on target (Mb) | 11216.66 | 9744.46 |
| Mapping rate on genome (%) | 99.81% | 99.84% |
| The average depth of sequencing | 185.92 | 161.52 |
| Capture specificity (%) | 59.72% | 61.85% |
| Duplicate rate on genome (%) | 11.37% | 12.50% |
| Fraction of target covered 1x (%) | 99.63% | 99.60% |
| Fraction of target covered 20x (%) | 98.19% | 97.34% |
| Fraction of target covered 50x (%) | 91.50% | 87.12% |
| Fraction of target covered 100x (%) | 71.03% | 62.20% |

MM = myopic maculopathy.

**Supplementary Table 3. Summary of the quality control filters across all individuals**

| A. SNP-level quality control for whole exome sequencing | | | |
| --- | --- | --- | --- |
| **Filters** | **Criteria** | **Removed** | **Removed (%)** |
|  |  |  |  |
| Depth for minor allele | Minor allele average depth <4x | 70,460 | 4.57% |
| Depth for site | Average depth <8x | 1,198,967 | 77.80% |
| VQSR | Filtered | 182,140 | 11.82% |
| 4x rate of minor allele | Minor allele depth <4 and rate <0.9 | 775,603 | 50.33% |
| 8x depth fraction of site | Case or control average depth < 8 and Call rate <0.9 | 1,020,664 | 66.23% |
| Homopolymer | Homopolymer runada ≥6 | 288,305 | 18.71% |
| Allelic balance | <0.25 or >0.75 | 56,629 | 3.67% |
| Mapping quality score | Root mean square <30 | 964 | 0.06% |
| Strand bias | FS>5 & SOR>3 | 8,495 | 0.55% |
| Hardy–Weinberg equilibrium test | P<10^-6^ | 4,483 | 0.29% |
| Removed |  | 1,541,074 | 100.00% |

| B. Sample-level quality control for whole exome sequencing | | |
| --- | --- | --- |
| **Filters** | **Criteria** | **Removed** |
| Mean sequencing depth | Fold coverage < 30x | 0 |
| Coverage | 10X coverage < 90% | 0 |
| GC content | Outliers | 1 |
| Mapping rate | Mapping read < 95% | 0 |
| IBD | 3rd-degree or closer relationship with other samples | 12 |
| Inbreeding coefficient | > 0.15 or < -0.15 | 1 |
| Principal Component Analysis | >6σ from the mean of any first 10 principal components | 0 |
| Sex | The coverage rate of chrY > 0.8 for male and <40% for female | 0 |
| All above filters | Any one | 13 |

**Supplementary Table 5. Gene-based analysis of low-frequency variants associated with high myopia and myopic maculopathy**

| **Gene** | **Case_**  **carrier** | **Case_**  **noncarrier** | **Control_**  **carrier** | **Control_**  **noncarrier** | **Fraction** | **OR** | **OR.**  **lower95** | **OR.**  **upper95** | **P value** |
| --- | --- | --- | --- | --- | --- | --- | --- | --- | --- |
| PRR35 | 12 | 391 | 0 | 294 | 0.017 | Inf | 2.066 | Inf | 0.002 |
| ATP2B4 | 30 | 373 | 7 | 287 | 0.053 | 3.293 | 1.391 | 9.011 | 0.003 |
| NFE2L3 | 22 | 381 | 4 | 290 | 0.037 | 4.179 | 1.398 | 16.857 | 0.004 |
| LAMA5 | 31 | 372 | 8 | 286 | 0.056 | 2.975 | 1.312 | 7.609 | 0.004 |
| COQ6 | 14 | 389 | 1 | 293 | 0.022 | 10.524 | 1.584 | 446.506 | 0.006 |
| LEPROTL1 | 25 | 378 | 6 | 288 | 0.045 | 3.170 | 1.248 | 9.573 | 0.009 |
| COQ7 | 9 | 394 | 0 | 294 | 0.013 | Inf | 1.457 | Inf | 0.012 |
| RTKN2 | 11 | 392 | 1 | 293 | 0.017 | 8.207 | 1.181 | 354.540 | 0.017 |
| TEX44 | 45 | 358 | 18 | 276 | 0.090 | 1.926 | 1.065 | 3.618 | 0.023 |
| ADGRA2 | 7 | 396 | 0 | 294 | 0.010 | Inf | 1.060 | Inf | 0.023 |
| C8A | 7 | 396 | 0 | 294 | 0.010 | Inf | 1.060 | Inf | 0.023 |
| GPNMB | 7 | 396 | 0 | 294 | 0.010 | Inf | 1.060 | Inf | 0.023 |
| KDR | 7 | 396 | 0 | 294 | 0.010 | Inf | 1.060 | Inf | 0.023 |
| PHLPP2 | 7 | 396 | 0 | 294 | 0.010 | Inf | 1.060 | Inf | 0.023 |
| RUBCNL | 7 | 396 | 0 | 294 | 0.010 | Inf | 1.060 | Inf | 0.023 |
| SPAG5 | 7 | 396 | 0 | 294 | 0.010 | Inf | 1.060 | Inf | 0.023 |
| STAT5A | 7 | 396 | 0 | 294 | 0.010 | Inf | 1.060 | Inf | 0.023 |
| BECN2 | 8 | 395 | 0 | 294 | 0.012 | Inf | 1.258 | Inf | 0.024 |
| CENPJ | 8 | 395 | 0 | 294 | 0.012 | Inf | 1.258 | Inf | 0.024 |
| EPHB1 | 8 | 395 | 0 | 294 | 0.012 | Inf | 1.258 | Inf | 0.024 |
| KIAA1549 | 8 | 395 | 0 | 294 | 0.012 | Inf | 1.258 | Inf | 0.024 |
| NEK4 | 8 | 395 | 0 | 294 | 0.012 | Inf | 1.258 | Inf | 0.024 |
| SLC10A5 | 8 | 395 | 0 | 294 | 0.012 | Inf | 1.258 | Inf | 0.024 |
| MANF | 18 | 385 | 4 | 290 | 0.032 | 3.385 | 1.099 | 13.893 | 0.026 |
| DNAJC10 | 10 | 393 | 1 | 293 | 0.016 | 7.439 | 1.048 | 324.180 | 0.030 |
| STK31 | 10 | 393 | 1 | 293 | 0.016 | 7.439 | 1.048 | 324.180 | 0.030 |
| SYTL1 | 10 | 393 | 1 | 293 | 0.016 | 7.439 | 1.048 | 324.180 | 0.030 |
| SLC25A32 | 30 | 373 | 10 | 284 | 0.057 | 2.282 | 1.064 | 5.323 | 0.031 |
| CAD | 13 | 390 | 2 | 292 | 0.022 | 4.858 | 1.087 | 44.674 | 0.031 |
| ARHGAP10 | 17 | 386 | 4 | 290 | 0.030 | 3.188 | 1.025 | 13.161 | 0.041 |
| UHRF1BP1 | 17 | 386 | 4 | 290 | 0.030 | 3.188 | 1.025 | 13.161 | 0.041 |
| RLF | 43 | 360 | 18 | 276 | 0.088 | 1.830 | 1.007 | 3.449 | 0.041 |
| ACOXL | 6 | 397 | 0 | 294 | 0.009 | Inf | 0.864 | Inf | 0.042 |
| ADGRL2 | 6 | 397 | 0 | 294 | 0.009 | Inf | 0.864 | Inf | 0.042 |
| CCT6A | 6 | 397 | 0 | 294 | 0.009 | Inf | 0.864 | Inf | 0.042 |
| CRISPLD1 | 6 | 397 | 0 | 294 | 0.009 | Inf | 0.864 | Inf | 0.042 |
| CTSZ | 6 | 397 | 0 | 294 | 0.009 | Inf | 0.864 | Inf | 0.042 |
| DIAPH1 | 6 | 397 | 0 | 294 | 0.009 | Inf | 0.864 | Inf | 0.042 |
| EPB42 | 6 | 397 | 0 | 294 | 0.009 | Inf | 0.864 | Inf | 0.042 |
| HGFAC | 6 | 397 | 0 | 294 | 0.009 | Inf | 0.864 | Inf | 0.042 |
| MKI67 | 6 | 397 | 0 | 294 | 0.009 | Inf | 0.864 | Inf | 0.042 |
| MTO1 | 6 | 397 | 0 | 294 | 0.009 | Inf | 0.864 | Inf | 0.042 |
| MYH13 | 6 | 397 | 0 | 294 | 0.009 | Inf | 0.864 | Inf | 0.042 |
| PC | 6 | 397 | 0 | 294 | 0.009 | Inf | 0.864 | Inf | 0.042 |
| PRKCSH | 6 | 397 | 0 | 294 | 0.009 | Inf | 0.864 | Inf | 0.042 |
| RARRES3 | 6 | 397 | 0 | 294 | 0.009 | Inf | 0.864 | Inf | 0.042 |
| RIBC2 | 6 | 397 | 0 | 294 | 0.009 | Inf | 0.864 | Inf | 0.042 |
| ROBO3 | 6 | 397 | 0 | 294 | 0.009 | Inf | 0.864 | Inf | 0.042 |
| SACS | 6 | 397 | 0 | 294 | 0.009 | Inf | 0.864 | Inf | 0.042 |
| USP42 | 6 | 397 | 0 | 294 | 0.009 | Inf | 0.864 | Inf | 0.042 |
| WDR11 | 6 | 397 | 0 | 294 | 0.009 | Inf | 0.864 | Inf | 0.042 |
| YY1AP1 | 6 | 397 | 0 | 294 | 0.009 | Inf | 0.864 | Inf | 0.042 |
| PRDX3 | 24 | 379 | 8 | 286 | 0.046 | 2.261 | 0.965 | 5.911 | 0.045 |

Deleterious (strict) set was applied for single-nucleotide variants function prediction that comprises missense variants annotated as “deleterious” by all five protein prediction algorithms, nonsense, splice-site, and indel frameshift variants. Fraction = The proportion of samples carrying the corresponding variant set of the gene in the all samples. OR = odds ratio.

**Supplementary Table 6. Primers’ information**

| A. Guide RNA and genotyping primers for knock-in mice (5' to 3') | | |
| --- | --- | --- |
| *Atp2b4*-p.P895S | guide RNA | CTCAACAGAGACTCGGTGGGGGG |
|  | Forward | AGTGGGTAGTGGGAGCTAGG |
|  | Reverse | ATGTGCTGAGACCACACAGG |
| *Lama5*-p.R1565C | guide RNA | GCCATAGAAGCCCGGGGCACAGG |
|  | Forward | TGTATCTGTCCACCACGCAC |
|  | Reverse | AGCTTCCAGAAAGCCCCATC |
| *Nfe2l3*-p.K580T | guide RNA | TTGAGTTTGCTCATTTTTAAGGG |
|  | Forward | TGAAGAACCAGATTCCGGGC |
|  | Reverse | GGCTTCCTTTTTGTGGCCTG |

| B. Primers for real-time quantitative PCR (5' to 3') | | |
| --- | --- | --- |
| *CD31* | Forward | ACGCTGGTGCTCTATGCAAG |
|  | Reverse | TCAGTTGCTGCCCATTCATCA |
| *CD34* | Forward | AAGGCTGGGTGAAGACCCTTA |
|  | Reverse | TGAATGGCCGTTTCTGGAAGT |
| *Cdh5* | Forward | CACTGCTTTGGGAGCCTTC |
|  | Reverse | GGGGCAGCGATTCATTTTTCT |
| *Nfe2l3* | Forward | TGAGCCAAGCTATAAGCCATGA |
|  | Reverse | AATGGTTCTTGTGCCTGTGAA |
| *Gapdh* | Forward | TGGATTTGGACGCATTGGTC |
|  | Reverse | TTTGCACTGGTACGTGTTGAT |
| *Actb* | Forward | GCAGGAGTACGATGAGTCCG |
|  | Reverse | ACGCAGCTCAGTAACAGTCC |
| *Hprt* | Forward | GTTGGGCTTACCTCACTGCT |
|  | Reverse | TAATCACGACGCTGGGACTG |
| *Rpl13a* | Forward | CCGGAAGCGGATGAATACCA |
|  | Reverse | GTTCACACCAGGAGTCCGTT |

**Supplementary Table 7. The quality control of the single-cell RNA sequencing data**

|  | ***Nfe2l3***  **p.-K580T** | **Wild-type** | **Average** |
| --- | --- | --- | --- |
| Number of reads | 312871128 | 303025251 | 307948790 |
| Sequencing saturation | 37.40% | 44.40% | 40.90% |
| Estimated number of cells | 15000 | 15000 | 15000 |
| Mean reads per cell | 20858 | 20202 | 20530 |
| Median genes per cell | 2242 | 2211 | 2227 |
| Total genes detected | 25282 | 25053 | 25168 |
| Median UMI counts per cell | 4810 | 4752 | 4781 |
| Outlier cells based on genes | 53 | 43 | 48 |
| Outlier cells based on mitochondrial contents | 6 | 21 | 14 |
| Outlier cells based on Doublets | 979 | 515 | 747 |
| Filtered cells | 1038 | 578 | 808 |

**Supplementary Table 10. The primary antibody for Western Blot**

| Protein | Vendor | Catalog number |
| --- | --- | --- |
| Tubulin | Abcam | ab6046 |
| COL1A1 | Abcam | ab260043 |
| α-SMA | Abcam | ab124964 |
| HIF-1α | CST | #36169 |
| CD31 | Abcam | ab222783 |
| CD34 | Abcam | ab81289 |
| CDH5 | Abcam | ab205336 |
| β-actin | Abcam | ab8227 |
| Myc | MBL | M047-A59 |
| FLAG | MBL | M185-7 |
